# On the Local Treatment of Carbuncle and Boil

**Published:** 1853

**Authors:** R. Flint

**Affiliations:** Consulting Surgeon to Stockport Infirmary


*On the Local Treatment of Carbuncle and Boil.*—By R. Flint, Esq., Consulting Surgeon to the Stockport Infirmary.

Considering these morbid actions as manifesting want of power in the vascular system of the parts affected, the author sought for an application that would, if possible; assist or increase it, and, at the same time, not interfere with the natural excretions from the skin. This he thought he saw in the common lead or litharge plaster, spread on the white leather of the shops. The former, if correctly prepared, will answer fully the object intended; and the latter ought to be well dressed, having no hard or harsh parts in what is selected for use. There is in lead plaster, besides its unirritating quality, a valuable property which seems to have been often overlooked or forgotten,—that of promoting or inducing perspiratory exhalation from the skin. Every surgeon must occasionally have noticed, that where the common lead plaster (containing no resin) has been allowed to remain on any part of the trunk or limbs for weeks, when it is removed there will be seen a considerable quantity of perspiratory fluid which has been pent up, showing no other consequences than a little soddening of the skin upon which it has been so long confined. For this reason, he always prefers using the plaster without any admixture of combination; and if fresh, it has every requisite adhesive property. To this peculiar effect on the cutaneous exhalants, he attributes much of its beneficial influence in the more acute boil or carbuncle. It must, however, be allowed, that in the extremely acute forms of this disease, the most soothing applications to the surface of the skin are indispensable, and that fomentations and poultices for twenty-four hours, or longer, may be quite necessary. As soon as the plaster can be borne, it ought to be applied without delay. It is likewise to be remembered, that there are idiosyncrasies were a plaster, entirely unirritating under ordinary circumstances, cannot be endured from the cutaneous irritation and excitement which follow its use. This, of course, is to be regarded as an exception to a rule.

The mode of application is the following:—After spreading the plaster in the ordinary manner, it is to be cut in dimensions according to the size of the carbuncle or boil to be dressed, carefully observing that it is large enough to include from half an inch to two or three inches of the surface around the tumor. Where this is large and deep, as in a carbuncle from four to six or eight inches in diameter, the circumference to be embraced should be proportionately large, and the leather, in such a case, shold be chosen soft and strong, in order to obtain an amount of adhesive mechanical support to the surrounding and subjacent parts, so as to enable them to originate and establish a new action. It must be well remembered, that it is not enough, in any of these dressings, to cut the plaster the exact size only of the tumor. It is expected that some of the surrounding surface should be taken in also.

In twenty-four hours after dressing, a favorable change in the appearance of the parts is generally discernible; and (what will be allowed to be a highly-important result, in those cases where the local irritation is fast inducing a state of cerebro-spinal distress and lesion, likely soon to end in coma and death) it will often, in a less period of time, afford a mitigation of suffering, which comes upon the patient like a charm, and restores him the rest and sleep to which he had been a stranger. If the tumor be prominent or pointing in any part, a crucial incision is to be cut in the plaster, and placed directly over it, that the discharge may have free egress; and should there be at first no suppuration, this mode of dressing quickly promotes it, and assists the living parts to detach and throw off, at the least possible loss, the sloughy cellular membrane. If the tumor is not disposed to slough or suppurate, the application decidedly hastens the resolution or absorption of the effused matter.

In a boil, the dressing is precisely the same as in a carbuncle, taking care that the plaster has sufficient hold of the tissues around, perhaps from half an inch to an inch. In a few hours the acute pain and suffering of the patient, which had unceasingly disturbed his rest and comfort, become greatly mitigated, and soon afterwards wholly removed; and the improvement in the condition of the parts is strikingly apparent on every renewal of the plaster. The little trouble and inconvenience of this method, both to patient and surgeon, strongly contrast with the inconvenience, wearisome labor, and attention required by the old plan; and whilst the latter necessarily confines the patient, even in minor cases, to the house, in bed, or on couch, the former will often be little or no restraint on his usual avocations.

It is requisite that the plaster be changed more or less frequently, according to the extent of discharge or slough; but the longer the interval, the more desirable, as the less the parts are disturbed the better. If there be much discharge or slough, soiling the cut edges of the plaster, it may be changed once a-day; if there be little, every third or fourth day will be sufficient; but the linen pledgets and bandage adjusted over the plaster, and absorbing the discharge as it exudes, may be replaced frequently. In general, no other dressing is required to the end of the treatment. The plaster must be reduced in its dimensions as the tumor lessens in size, whether it be by suppuration, slough, or absorption. In some instances, towards the close, simple or calamine serate, or the strong solution of nitrate of silver (qr. to half oz.), may be desirable.

From what he has seen of this method of treatment in a number of patients during the last several years, and from the speedy relief and total removal of pain and distress in the worst cases, and in bad temperaments, the author looks upon it as of great value, if attentively carried out; and he states his persuasion that it will render recourse either to the knife, to caustic remedies, or any of the other more severe measures of our art, unnecessary.

*Dublin Medical Press*, July 20, 1853.

